# Invasive Enterovirus and Parechovirus Infections in Adults, Alberta, Canada, 2023–2025

**DOI:** 10.3201/eid3211.260941

**Published:** 2026-11

**Authors:** Jamil N. Kanji, Hong Yuan Zhou, Nathan Zelyas, Kanti Pabbaraju, Anita Wong, Kara Gill, Tarah Lynch, Graham Tipples, Nabeela Nathoo, Dima Kabbani, Adil Adatia

**Affiliations:** Alberta Precision Laboratories Public Health Laboratory, Edmonton, Alberta, Canada (J.N. Kanji, H.Y. Zhou, N. Zelyas, K. Pabbaraju, A. Wong, K. Gill, T. Lynch, G. Tipples); University of Calgary, Calgary, Alberta, Canada (J.N. Kanji, H.Y. Zhou, T. Lynch); University of Alberta, Edmonton (J.N. Kanji, N. Zelyas, G. Tipples, N. Nathoo, D. Kabbani, A. Adatia)

**Keywords:** enterovirus, parechovirus, ocrelizumab, B-cell depletion therapy, hypogammaglobulinemia, immunocompromised host, pharmacovigilance, meningitis/encephalitis, viruses, Alberta, Canada

## Abstract

We conducted a retrospective, population-based laboratory surveillance study during May 2023–December 2025 in Alberta, Canada, to characterize adult invasive enterovirus and parechovirus infections and to identify groups at high risk for those infections. Of 15,621 samples tested, 162 (1.0%) were virus positive, of which 51 (31.5%) samples from 39 patients indicated invasive infections. Cerebrospinal fluid, blood, and cardiac tissue were the most frequent virus-positive specimens. The invasive enterovirus and parechovirus incidence rate was 0.37 cases/100,000 person-years; rates were higher in patients <50 versus >50 years of age (incidence rate ratio 8.2; p<0.001). The annual incidence rates were 0.20–0.56 cases/100,000 person-years; rates appeared higher in 2025 than in 2024 (incidence rate ratio 2.1; p = 0.11). Severe disseminated enterovirus infection occurred in 2 patients who received ocrelizumab. Continuous surveillance and ongoing optimization of enterovirus and parechovirus detection assays is essential for comprehensive public health tracking.

Enteroviruses and parechoviruses are globally circulating picornaviruses that typically cause mild or asymptomatic infections but can cause severe invasive disease in certain clinical contexts. Several nationwide studies in Denmark reported that enteroviruses are among the leading viral causes of adult meningitis, which has a favorable outcome in immunocompetent patients (*1*–*3*). Beyond meningitis, enteroviruses can cause encephalitis and meningoencephalitis (*4*), as well as myocarditis and pericarditis (commonly caused by coxsackievirus B) (*5*). Invasive enterovirus infection can also manifest a disseminated sepsis-like syndrome in immunocompromised hosts (*6*). Surveillance and cerebrospinal fluid (CSF)–based typing studies in Europe and North America have characterized coxsackievirus B5, enterovirus A71 (EV-A71), and EV-D68 as commonly identified invasive genotypes (*7*–*9*). Among echoviruses (a subset of enteroviruses), echoviruses 6, 9, 11, and 18 are commonly reported invasive types (*7*,*10*). Invasive enterovirus-type distribution likely varies according to clinical syndrome, geography, and time, which reflects the heterogeneity of observed genotypes (*11*).

Parechovirus infections are reported less frequently in adults than in neonates and infants. One study found parechovirus prevalence in adults was 0.18% (*12*). Nevertheless, adult cases of parechovirus-induced meningoencephalitis and severe systemic infections have been documented, usually in the setting of immunosuppression or critical care (*12*). That finding suggests that adult disease might be underrecognized. Parechoviruses 1, 3, 5, and 6 are the most frequently reported invasive parechovirus genotypes (*12*–*15*). Outcome data for adults with invasive parechovirus infections are limited to small series and case reports that describe outcomes ranging from recovery to fatal encephalitis in vulnerable hosts (*16*).

Being immunocompromised is commonly reported as a risk factor for invasive enterovirus or parechovirus infections in adults (*17*). Severe enterovirus infections have been described in patients who have hematologic malignancies, organ transplantation, and hypogammaglobulinemia (congenital or acquired) and in those receiving B-cell depleting therapies (*17*–*19*). Long-term follow-up studies on enterovirus infections of the central nervous system (CNS) in adults also identified immunosuppression as a predictor of poor outcomes (*20*). More recently, multiple reports have described disseminated enterovirus infections in patients who had B-cell depletion after ocrelizumab treatment for multiple sclerosis; almost all patients had documented hypogammaglobulinemia, and outcomes included recovery, residual deficits, and death (*9*,*21*–*24*).

Data on invasive enterovirus and parechovirus infections in adults are limited, particularly in immunocompromised populations. Prompted by the laboratory identification of severe disseminated enterovirus infections in ocrelizumab-treated patients, we conducted a population-based review of laboratory-confirmed invasive enterovirus and parechovirus infections among adults in Alberta, Canada, during 2023–2025.

## Materials and Methods

### Study Design, Data Extraction, and Chart Review

We extracted laboratory data regarding all enterovirus and parechovirus testing in adult patients conducted in Alberta, Canada, during May 1, 2023–December 31, 2025, from the provincial electronic medical records in the laboratory information system (Epic, https://www.epic.com). The Conjoint Health Research Ethics Board at the University of Calgary in Calgary, Alberta, Canada, approved this study before data collection (study identification no. REB25-2031).

We included data for adult patients (>17 years of age at the time of specimen collection, according to the provincial health administration definition). We defined invasive infection as the detection of enterovirus or parechovirus nucleic acid by using real-time reverse transcription PCR of blood, CSF, joint fluid, or tissue biopsy samples collected from a sterile site. We did not classify detection of enteroviruses or parechoviruses in tissue samples from skin, respiratory, oral, or mucosal tissue as an invasive infection.

We extracted and tabulated data for specimens that met the inclusion criteria (samples from adult patients and invasive infection). We conducted retrospective chart reviews to collect patient information regarding disease manifestation, diagnostic evaluation, presence of underlying immunosuppressive conditions or medications, need for critical care, extracorporeal membrane oxygenation, and whether the patient survived the infection. We categorized patients as either having no recognized immunosuppression, receiving ocrelizumab, or having a recognized immunocompromising condition. We defined geographic distribution by using the second digit within the patient’s postal code, which categorized the patient as residing in urban or rural postal sorting areas. We used a composite of intensive or coronary care unit admission, requirement for extracorporeal membrane oxygenation, or death attributable to enterovirus or parechovirus infection to define a critical or fatal outcome. We recorded results of genetic testing for inborn errors of immunity when those tests were requested by clinicians managing each patient.

### Setting and Enteroviruses and Parechoviruses Testing

As of April 1, 2026, Alberta had a population of 5.06 million persons (https://economicdashboard.alberta.ca/dashboard/population-quarterly). Testing for enterovirus and parechovirus in Alberta is conducted centrally at the Alberta Precision Laboratories Public Health Laboratory in Calgary. Testing is performed on a wide range of specimen types by using a validated, nonquantitative, laboratory-developed multiplex real-time reverse transcription PCR that targets the 5′ nontranslated regions of both enteroviruses and parechoviruses (Appendix) (*25*).

### Typing of Enterovirus Isolates

We performed enterovirus typing for specimens from all invasive cases. For patients with multiple positive specimens, we selected the specimen that had the lowest cycle threshold (Ct) value for genotyping. Genotyping included PCR amplification and Sanger sequencing of a segment of the 5′ nontranslated region (293 bp), the viral protein (VP) 4 gene (207 bp), and 5′ region of the VP2 gene (250 bp) (*26*,*27*). We performed phylogenetic analysis of 62 amino acids from the VP2 protein by using the neighbor-joining method in MEGA version 10.2.2 (https://www.megasoftware.net) (Appendix).

### Statistical Analysis

We performed statistical analyses by using R Studio version 2026.08.0, build 187 (Posit Software, https://posit.co). We compared Ct values by using medians and interquartile ranges and by using the Mann-Whitney U test for nonnormal distributions.

We obtained population estimates for persons in Alberta who were >17 years of age from the Government of Alberta Office of Statistics and Information and stratified those data according to age and sex during 2023–2025. Only two thirds of a population year were available for estimates in 2023 because surveillance commenced in May (hence, 2023 denominator contributions were multiplied by 2/3). We used those estimates to calculate incidence rates for all invasive enterovirus and parechovirus infections, enterovirus-only infections, calendar year, age strata (17–29, 30–49, 50–64, and >65 years of age), and sex, as well as for critical or fatal invasive infection. We expressed all rates as per 100,000 person-years along with exact 95% Poisson CIs because of the low number of events.

The manufacturer of ocrelizumab (Hoffman-La Roche Canada, https://www.rochecanada.com) provided the number of patients who received >1 dose of the agent in Alberta annually during 2023–2025. We estimated the crude risk for invasive enterovirus disease and mortality rates for patients taking ocrelizumab by dividing the number of observed events by the treated patient-years (summed annual counts of active ocrelizumab-treated persons) and calculated exact 95% CIs as described. We prorated the 2023 patient count for the May–December observation period as described. We scaled the results according to treated patient-years. We assumed that invasive enterovirus disease was a patient-level outcome rather than an infusion-level outcome and that stable treatment exposure occurred within each calendar year (i.e., dosing every 6 months).

We assessed event rates between groups by calculating pairwise incidence rate ratios (IRRs) and exact 95% Poisson CIs. To evaluate rate differences according to age, we tested for consistency across all age groups (exact conditional test of homogeneity) assuming a null hypothesis of equal incidence. We compared incidences between sex, 2024 and 2025, and various age categories (Appendix).

## Results

### Virus Testing

Over the ≈3-year study period, a total of 15,621 samples were received for enterovirus and parechovirus testing (Table 1). Of those, 162 (1.0%) samples were positive; 51 (31.5%) met our definition of invasive infection. CSF was the most common specimen type received (40 [78.4%] samples), followed by blood (8 [15.7%] samples) and heart tissue (3 [5.9%] samples) (Appendix Table 1). Of the 51 samples positive for invasive infection, 50 were positive for enterovirus and 1 for parechovirus (Appendix).

**Table 1:** Numbers of specimens tested for enterovirus and parechovirus from adults with invasive infections, Alberta, Canada, 2023–2025

Specimens	No. (%) specimens/study year			Total, no. (%)
	2023	2024	2025	
No. specimens received*	3,723	5,767	6,131	15,621
Positive for virus	42 (1.1)	58 (1.0)	62 (1.0)	162 (1.0)
Positive invasive infection†	15 (35.7)	9 (15.5)	27 (43.5)	51 (31.5)
Cerebrospinal fluid samples	15 (100)	8 (88.9)	17 (63.0)	40 (78.4)
Blood samples	0	1 (11.1)	7 (25.9)	8 (15.7)
Sterile tissue samples	0	0	3 (11.1)	3 (5.9)

*All specimens received (sterile and nonsterile sites). †No. (%) virus-positive samples. Counts reflect positive specimens; some patients contributed >1 specimen.

### Characteristics of Patients with Invasive Virus Infection

The 51 positive specimens were from 39 patients who were a median age of 32.5 years (range 17–72 years; interquartile range 23–35 years); 22 (56.4%) were female and 17 (43.6%) male (Table 2; Appendix Tables 2, 3). According to postal code sorting, 33 (84.6%) patients resided in urban areas and 6 (15.4%) in rural areas. Invasive cases occurred in 14 (35.9%) patients in 2023, eight (20.5%) in 2024, and 17 (43.6%) in 2025 (38 cases were caused by enterovirus; 1 was caused by parechovirus in 2024). Most (69.2%; n = 27) patients were hospitalized; median stay was 3 days. Only 2 patients required admission to a critical care setting. The most common enterovirus syndromes were aseptic meningitis (31 [79.5%] patients) and encephalitis or meningoencephalitis (6 [15.4%] patients). Two patients in the cohort died. 

**Table 2:** Clinical characteristics of patients in study of invasive enterovirus and parechovirus infections in adults, Alberta, Canada, 2023–2025*

Characteristics	Not IC, n = 33	IC by ocrelizumab, n = 3	IC for other reasons, n = 3	Total
Median age, y (IQR)	31 (23–35)	29 (29–32)	39 (38–53)	32.5 (23–35)
Age groups, y				
17–29	14 (42.4)	2 (66.7)	0	16 (41.0)
30–49	17 (51.5)	1 (33.3)	2 (66.7)	20 (51.3)
50–64	1 (3.0)	0	0	1 (2.6)
>65	1 (3.0)	0	1 (33.3)	2 (5.1)
Study year				
2023, May–December	14 (42.4)	0	0	14 (35.9)
2024	7 (21.2)	1 (33.3)	0	8 (20.5)
2025	12 (36.4)	2 (66.7)	3 (100)	17 (43.6)
Sex				
F	18 (54.5)	2 (66.7)	2 (66.7)	22 (56.4)
M	15 (45.5)	1 (33.3)	1 (33.3)	17 (43.6)
Geographic distribution†				
Urban postal code	29 (87.9)	1 (33.3)	3 (100)	33 (84.6)
Rural postal code	4 (12.1)	2 (66.7)	0	6 (15.4)
Enterovirus/parechovirus syndrome				
Aseptic meningitis‡	28 (84.8)	0	3 (100)	31 (79.5)
Encephalitis/meningoencephalitis	5 (15.2)	1 (33.3)	0	6 (15.4)
Disseminated infection, myocarditis	0	2 (66.7)	0	2 (5.1)
Clinical course and outcomes				
Hospitalized	21 (63.6)	3 (100)	3 (100)	27 (69.2)
Median length of stay, d (IQR) [range]	2 (2–3) [1–11]	45 (26.5–62.5) [8–80]§	4 (3.5–5.5) [3–7]	3 (2–5.5)
ICU or ECMO admission	0	2 (66.7)	0	2 (5.1)
Death	0	2 (66.7)	0	2 (5.1)
Underlying illnesses				
None	10 (30.3)	0	0	10 (25.6)
Hyperlipidemia	1 (3.0)	0	1 (33.3)	2 (5.1)
Hypothyroid	2 (6.1)	0	1 (33.3)	3 (7.7)
Inflammatory bowel disease	0	0	1 (33.3)	1 (2.6)
Irritable bowel syndrome	0	1 (33.3)		1 (2.6)
Lymphoma	0	0	1 (33.3)	1 (2.6)
Migraine	4 (12.1)	0	0	4 (10.3)
Multiple sclerosis	0	3 (100)	0	3 (7.7)
Obstructive sleep apnea	0	0	1 (33.3)	1 (2.6)
Opioid/substance use disorder	4 (12.1)	0	0	4 (10.3)
Solid organ transplant	0	0	1 (33.3)	1 (2.6)
Rheumatoid arthritis	0	1 (33.3)	0	1 (2.6)

*Values are no. (%) except as indicated. Total no. patients = 39. ADHD, attention deficit hyperactivity disorder; ECMO, extracorporeal membrane oxygenation; IC, immunocompromised; ICU, intensive care unit; IQR, interquartile range. †Urban and rural postal code classifications were according to the second character of the Forward Sortation Area of Canada Post; numerals 1–9 were classified as urban and 0 as rural. Those categories represent postal delivery geography and do not necessarily correspond to census-based definitions of urban and rural residence (https://www.canadapost-postescanada.ca/cpc/en/support/articles/addressing-guidelines/postal-codes.page, accessed 2026 Aug 10). ‡Thirty (96.8%) of 31 patients with aseptic meningitis had cerebrospinal fluid pleocytosis (leukocyte count >5 × 10^6^ cells/L). §One of the 3 hospitalized patients in this group had 2 hospitalizations, which were counted separately.

CSF analysis was conducted for 37 (94.9%) patients (Appendix Table 3). All patients with aseptic meningitis had CSF leukocytosis (>5 × 10^6^ leukocytes/L). In 30 (76.9%) patients who underwent CNS imaging, 28 (93.3%) had no acute abnormality reported. The cases demonstrated a seasonal pattern (Figure 1) in 2023 and 2025, with peaks in the summer and early autumn; fewer cases were reported in 2024.

**Figure 1:**
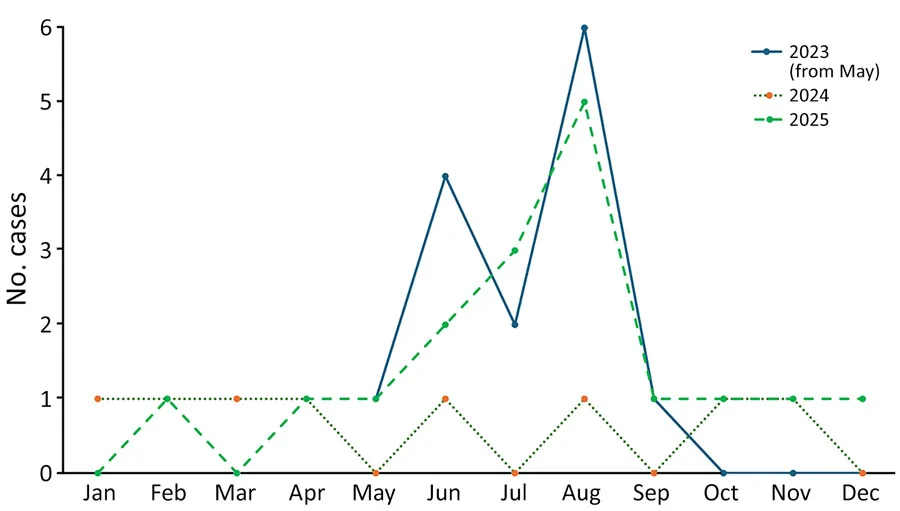
Seasonal variation of invasive enterovirus and parechovirus infections in adults, Alberta, Canada, 2023–2025.

### Incidence Rates and Comparisons

The overall incidence rate of invasive enterovirus and parechovirus infections was 0.37 (95% CI 0.27–0.51) cases/100,000 person-years (Table 3). Annual incidence rates varied from 0.20 to 0.56 cases/100,000 person-years; the highest incidence was observed in 2023 and lowest in 2024. Incidence varied significantly according to age group (overall p value was <0.001), and the highest rates were among adults 17–29 and 30–49 years of age. The incidence rate for a critical or fatal outcome was 0.02 (95% CI 0.0–0.07) cases/100,000 person-years. Analysis combining age strata demonstrated that the incidence among adults <50 years of age was 8.2-fold higher than among those >50 years of age (IRR 8.21 [95% CI 2.59–41.65]; p<0.001). Incidence did not differ significantly according to sex (IRR 1.30 [95% CI 0.66–2.61]; p = 0.43). Incidence appeared to be approximately 2-fold higher in 2025 than in 2024, although that difference was not statistically significant (IRR 2.07 [95% CI 0.85–5.54]; p = 0.11).

**Table 3:** Population-estimated incidence rates and rate comparisons for invasive enterovirus and parechovirus infections in adults, Alberta, Canada, 2023–2025*

Measurements†	Incidence/100,000 PY (95% CI)‡	IRR (95% CI)‡	p value
Overall incidence rates			
Invasive enterovirus and parechovirus	0.37 (0.27–0.51)	NA	
Enterovirus only	0.36 (0.26–0.50)	NA	
Incidence rates according to age group, y			
17–29	0.72 (0.41–1.16)	NA	
30–49	0.51 (0.31–0.78)	NA	
50–64	0.05 (0.00–0.25)	NA	
>65	0.10 (0.01–0.36)	NA	
Age-group comparisons, y§			
17–29 vs. 30–49	NA	1.42 (0.69–2.89)	0.30
<50 vs. >50	NA	8.21 (2.59–41.65)	<0.001
Annual incidence rates¶			
May–December 2023	0.56 (0.31–0.95)	NA	
2024	0.20 (0.09–0.40)	NA	
2025	0.42 (0.25–0.68)	NA	
Annual comparison			
2025 vs. 2024	NA	2.07 (0.85–5.54)	0.11
Sex-stratified incidence rates#			
F	0.42 (0.26–0.64)	NA	
M	0.33 (0.19–0.52)	NA	
Sex comparison			
F vs. M	NA	1.30 (0.66–2.61)	0.43
Critical or fatal outcome incidence rate**			
ICU/CCU admission, ECMO support, or death attributed to invasive virus infection	0.02 (0.00–0.07)	NA	

*CCU, cardiac care unit; ECMO, extracorporeal membrane oxygenation; ICU, intensive care unit; IRR, incidence rate ratio; NA, not applicable; PY, person-years. †Unless otherwise indicated, rates were calculated for the entire study (May 2023–December 2025) and represent invasive enterovirus and parechovirus infections. Rates were calculated by using population estimates obtained from the Alberta Population Table, Government of Alberta, Office of Statistics and Information (https://goa-osi-dashboards.azurewebsites.net/Reports). ‡Exact Poisson 95% CI was calculated. §The overall comparison of incidence rates across all 4 age groups was statistically significant (exact conditional test of homogeneity; p<0.001). ¶Because the surveillance period in this study began in May 2023, the annualized 2023 incidence disproportionately represents months of expected seasonal enterovirus circulation and should not be directly compared with complete calendar years. #Reported in the patient’s charts at the time PCR was used to detect enterovirus and parechovirus. **Includes all patients infected with invasive enterovirus or parechovirus (n = 39).

### Invasive Enterovirus Infections in Immunocompromised Patients

Six (15.4%) of 39 patients with invasive enterovirus infection were considered immunocompromised (Table 2; Appendix Table 4). The underlying causes of immunosuppression were CD20 monoclonal antibody treatment in 4 patients (3 used ocrelizumab for multiple sclerosis, and 1 used rituximab for lymphoma), organ transplantation in 1 patient, and infliximab use for Crohn’s disease in 1 patient. Three of the 6 patients had aseptic meningitis, 1 had encephalitis, and 2 had disseminated infection with fulminant myocarditis. The 2 patients with myocarditis were receiving ocrelizumab, and both died (Appendix Table 4).

The numbers of patients who received >1 dose of ocrelizumab in Alberta during the study years were 1,778 for 2023, 1,972 for 2024, and 2,119 for 2025 (M. Davies, Hoffman-La Roche Limited, pers. comm., email, 2026 Jan 14). On the basis of those data, the estimated crude incidence rate for invasive enterovirus disease among persons receiving ocrelizumab was 56.9 (95% CI 11.7–166.2) cases/100,000 ocrelizumab-treated patient-years. The crude incidence rate for death caused by invasive enterovirus disease for a person using ocrelizumab was 37.9 (95% CI 4.6–136.9) cases/100,000 ocrelizumab-treated patient-years. Denominators for other immunocompromised populations in the province were not available, preventing subgroup incidence determination.

### Genotyping of Invasive Enterovirus Isolates

Invasive enteroviruses could be amplified for genotyping in 18 (46.2%) of 39 cases (Figure 2; Appendix Table 5). The most frequently identified enterovirus was echovirus 30, followed by coxsackievirus B4 and echovirus 9. Echovirus 9 and echovirus 18 were observed over multiple surveillance years (both during 2024 and 2025). Enterovirus typing was possible for samples from 4 of 6 immunocompromised patients (Appendix Table 4). Coxsackievirus B3 was identified in 2 of 6 immunocompromised patients who had sepsis and myocarditis (both receiving ocrelizumab), echovirus 9 was found in 1 patient with meningitis, and EV-A71 was found in 1 patient with meningoencephalitis. Both fatal cases involved coxsackievirus B3 that was associated with severe, fulminant myocarditis and heart failure (Appendix Table 4). We submitted all 18 sequences from this study to GenBank (accession nos. PZ914341–58) (Appendix Table 6).

**Figure 2:**
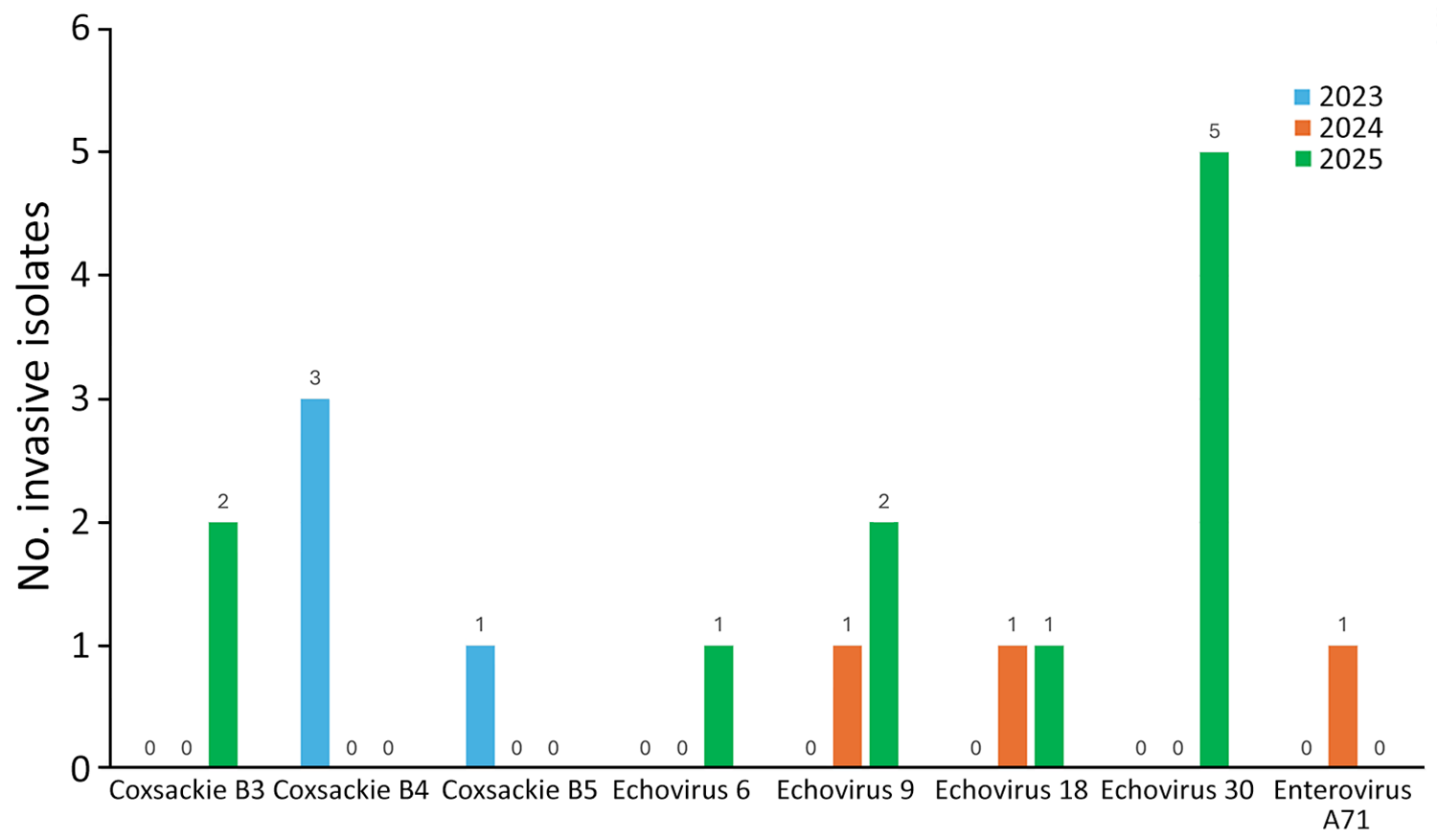
Genotyping of invasive enterovirus isolates in study of invasive enterovirus and parechovirus infections in adults, Alberta, Canada, 2023–2025. Of 39 unique patients with invasive virus infections, PCR amplification and genotyping were performed on samples from 4 of 14 patients in 2023, three of 8 patients in 2024, and 11 of 17 patients in 2025.

We created a dendrogram to evaluate the relatedness of the typed strains, including strains that infected ocrelizumab-treated persons (Appendix Figure). Phylogenetic analysis of the VP2 regions demonstrated that clinical isolates from adult patients clustered among established reference strains without evidence of close relatedness among the sequences. Those findings indicate genetic diversity among the enteroviruses causing invasive disease in Alberta without a single clonal cluster.

## Discussion

In a provincewide, laboratory-based retrospective study in Alberta, Canada, we identified 39 adults with invasive enterovirus or parechovirus infections over a 32-month period, which resulted in an overall incidence rate of 0.37 cases/100,000 person-years. Most patients were not immunosuppressed and had favorable outcomes; aseptic meningitis was the principal manifestation of infection. Deaths were only recorded among a subgroup of immunocompromised patients who received ocrelizumab, a CD20-binding B-cell depleting agent.

The incidence of invasive enterovirus infection in our cohort was higher among adults <50 years of age. Rates were 0.51–0.72 cases/100,000 person-years for persons 17–49 years of age, compared with 0.05–0.10 cases/100,000 person-years for those >50 years of age. That age distribution is consistent with several studies in Denmark that identified a median age of 31–33 years among adults with invasive enterovirus infection (*1*,*3*). In our analysis, incidence was ≈8-fold higher among adults <50 years than for those >50 years of age; however, the analysis might be limited by low case numbers in the >50 years of age group. Although the exact reason for the ≈8-fold difference is unclear, published studies hypothesized that older adults are protected more by accumulated type-specific and cross-reactive immunity than younger adults (*1*,*28*). In contrast, the observed incidence did not differ according to sex, suggesting that age was the more prominent variable associated with invasive infection, as previously reported (*1*,*3*). However, 2 studies identified sex as a driver of outcome; 1 of those studies reported female patients with invasive enterovirus infections were 2.3 times more likely to experience an unfavorable outcome at discharge than those who had other causes of viral meningitis (*1*,*3*).

The annual incidence of invasive enterovirus infections varied during the surveillance period. Fewer infection cases were identified in 2024 than in 2025, which is unlikely to represent an interruption in laboratory surveillance. We can confirm that laboratory surveillance was continuous throughout 2024, and the overall enterovirus and parechovirus PCR test positivity remained at 1% across study years. Although the population incidence rate of invasive enterovirus infection appeared ≈2-fold higher in 2025 (0.42 cases/100,000 person-years) than in 2024 (0.20 cases/100,000 person-years), that difference was not statistically significant (IRR 2.1; p = 0.11). Because of the small number of invasive infections, we hypothesize that the observed annual differences reflect year-to-year variation in enterovirus circulation rather than a sustained change in incidence. The 2023 rate was not directly compared (in terms of IRRs) to 2024 and 2025 because surveillance only started in May of that year.

Among patients receiving ocrelizumab in our cohort, 3 invasive enterovirus infections occurred, which caused 2 deaths. Although we reported few deaths (thus, requiring cautious interpretation), that clustering highlights a potential pharmacovigilance signal. The crude incidence rate of 56.9 cases/100,000 ocrelizumab-treated patient-years in our study is higher than reported population-based enterovirus-mediated meningitis estimates (1.8–3.6 cases/100,000 patient-years) (*1*), although differences in study populations and endpoints make direct comparisons difficult. Disseminated enterovirus infection among patients receiving ocrelizumab has been previously reported (Appendix Table 7), and numerous recent reports have also emphasized a potential safety signal (*21*,*22*,*24*). The mechanisms leading to infection have been proposed to be ocrelizumab-induced hypogammaglobulinemia that causes deficient humoral immunity and B-cell depletion that causes reduced enterovirus clearance. Two of the 3 affected patients in our study had serum IgG concentrations above the commonly cited 4 g/L threshold for immunoglobulin replacement and had no history of recurrent infections (*29*). Clinician-ordered testing for inborn errors of immunity was performed for only 2 ocrelizumab-treated patients; 1 of those patients had a *TNFRSF13B* C104R gene variant, which increases susceptibility to antibody deficiency (*30*). However, because the remaining patients in this cohort (including other immunocompromised patients) were not tested, the potential contribution of underlying genetic susceptibilities to severe enterovirus disease could not be assessed. Further study will be needed to determine clinical predictors of susceptibility in this group of patients.

Our genotyping results suggest that multiple enterovirus types (e.g., coxsackievirus B viruses, various echoviruses, and EV-A71) circulated during the study period. The frequent detection of echovirus 9 and echovirus 30 is consistent with previous reports identifying those as common virus types that contribute to adult aseptic meningitis (*31*). The seasonal distribution of cases mirrors that of established enterovirus circulation patterns in North America, which peak during late spring through early autumn (*32*).

Our findings highlight critical diagnostic considerations pertaining to enterovirus infection diagnoses. Neuroimaging showed no obvious abnormality in 28 (93.3%) of 30 patients who were imaged, consistent with previous studies of virus-induced or aseptic meningitis in which leptomeningeal enhancement is variably present (*1*,*33*). Many available multiplex respiratory panels combine enterovirus and rhinovirus targets. Although those panels can provide early indicators of enterovirus disease, distinction from rhinovirus is required, and the presence of enterovirus in respiratory samples alone cannot establish invasive, disseminated disease (*34*). Furthermore, the enterovirus load within the CNS is typically low (*35*). Low-level smoldering enterovirus loads in immunocompromised patients who have potential meningoencephalitis have been described (*36*). Thus, in immunocompromised patients who have delayed or atypical manifestations of infection, testing additional specimens, such as blood, other sterile sites, or tissue (e.g., myocardial biopsy), should be considered to enhance the diagnostic yield (*37*,*38*).

The principal strength of this study is the centralized testing of enterovirus from different specimen types; thus, we captured all positive samples accurately during the study period. The first limitation of our study is that the genotyping of enterovirus isolates was only available for 18 (46.2%) of 39 isolates because of low virus loads. Our sequencing assay does not reliably amplify virus sequences when the Ct is >34 cycles. Second, because a retrospective laboratory-based study relies on clinician-directed testing, we were more likely to capture more severe infections. Patients with milder cases might not have sought care or undergone diagnostic testing. Finally, the risk estimates for ocrelizumab-treated patients should be interpreted with caution because of the small number of events and reliance on aggregate measures of drug treatment exposure. Further study will be critical to help refine those estimates.

In conclusion, invasive adult enterovirus and parechovirus infections are uncommon in Alberta and manifest most often as self-limited aseptic meningitis among adults <50 years of age. Severe infections that required care in an intensive care unit or had a poor outcome were rare and occurred exclusively among ocrelizumab-treated patients. Maintaining a high index of suspicion for disseminated disease in high-risk patients might expedite earlier diagnosis and improve outcomes. Ongoing surveillance and broader optimization of enterovirus and parechovirus detection assays remain essential for comprehensive public health tracking.

Appendix Additional information for invasive enterovirus and parechovirus infections in adults, Alberta, Canada, 2023–2025.
