# Supplementary material for: Invasive Enterovirus and Parechovirus Infections in Adults, Alberta, Canada, 2023–2025
Source: Emerg Infect Dis. 2026 Nov;32(11):1846–54. doi: 10.3201/eid3211.260941 (PMC13715878; doi:10.3201/eid3211.260941)
Supplement: Supplementary file 1 [file 26-0941-Techapp-s1.pdf]

Article DOI: https://doi.org/10.3201/eid3211.260941
EID cannot ensure accessibility for supplementary materials supplied by authors. Readers who have difficulty accessing supplementary content should contact the authors for assistance.

Invasive Enterovirus and Parechovirus Infections in Adults, Alberta, Canada, 2023–2025

Appendix

Additional Methods

Further Details for EV/PeV rRT-PCR Testing

Testing for enterovirus and parechovirus at the Alberta Precision Laboratories, Public Health Laboratory, Calgary site, Canada, was done by using a laboratory-developed multiplex real time reverse transcription PCR (rRT-PCR) targeting the 5′-nontranslated regions of both enterovirus and parechovirus. The test is qualitative (i.e., does not generate a quantifiable virus load) and validated for detection of enterovirus and parechovirus from cerebrospinal fluid, feces, amniotic fluid, lesion swab samples, oral swab samples, respiratory specimens, plasma, serum, and tissue samples. Specimens first underwent nucleic acid extraction by using EMAG or EasyMag kits (bioMérieux, https://www.biomerieux.com) and then underwent rRT-PCR for enterovirus and parechovirus detection. Any cycle threshold (Ct) value that detected virus in <u><</u>41 cycles was considered a positive result. The rRT-PCR was repeated in duplicate on normally sterile samples that had a Ct value of 35–41 to ensure there was detectable virus. Further details on the validation of this assay can be found in this reference (*1*).

Further details regarding phylogenetic tree construction.

Evolutionary history was inferred by using the neighbor-joining method (*2*). The optimal tree with the sum of branch length = 1.02446764 is shown (Appendix Figure). The percentage of replicate trees in which the associated taxa clustered together in the bootstrap test (100 replicates) are shown next to the branches (*3*). The tree was drawn to scale and has branch lengths in the same units as those of the evolutionary distances used to infer the phylogenetic

tree. The evolutionary distances were computed by using the Poisson correction method (*4*) and are in the units of the number of amino acid substitutions per site. This analysis involved 36 aa sequences. All positions containing gaps and missing data were eliminated (complete deletion option). There were a total of 54 positions in the final dataset. Evolutionary analyses were conducted in MEGA X (*5*).

Additional Results

Cycle Threshold (Ct) Results

The median (interquartile range [IQR] Q1–Q3) Ct values according to specimen type for the positive enterovirus samples were 34.4 (IQR 33.4–36.1) cycles for cerebrospinal fluid (CSF), 32.5 (27.1–36.6) cycles for blood, and 31.5 (29.2–34.7) cycles for heart tissue. The Ct value for the CSF sample positive for parechovirus was 31.0 cycles. Comparison of Ct values for enterovirus testing between CSF and blood samples did not show values differed significantly ($p = 0.33$).

References

1. Pabbaraju K, Wong S, Wong AA, Tellier R. Detection of enteroviruses and parechoviruses by a multiplex real-time RT-PCR assay. Mol Cell Probes. 2015;29:81–5. PubMed https://doi.org/10.1016/j.mcp.2015.02.001
2. Saitou N, Nei M. The neighbor-joining method: a new method for reconstructing phylogenetic trees. Mol Biol Evol. 1987;4:406–25. PubMed https://doi.org/10.1093/oxfordjournals.molbev.a040454
3. Felsenstein J. Confidence limits on phylogenies: an approach using the bootstrap. Evolution. 1985;39:783–91. PubMed https://doi.org/10.1111/j.1558-5646.1985.tb00420.x
4. Zuckerandl E, Pauling L. Evolutionary divergence and convergence in proteins. In: Bryson V, Vogel HJ, editors. Evolving genes and proteins. New York: Academic Press; 1965. p. 97–166.
5. Kumar S, Stecher G, Li M, Knyaz C, Tamura K. MEGA X: molecular evolutionary genetics analysis across computing platforms. Mol Biol Evol. 2018;35:1547–9. PubMed https://doi.org/10.1093/molbev/msy096

6. Nicolini LA, Canepa P, Caligiuri P, Mikulska M, Novi G, Viscoli C, et al. Fulminant hepatitis associated with echovirus 25 during treatment with ocrelizumab for multiple sclerosis. JAMA Neurol. 2019;76:866–7. PubMed https://doi.org/10.1001/jamaneurol.2019.0522

7. Chaggar G, Waldner D, Scozzafava J, Harrison R, Kashmere J, Jassal R, et al. Teaching neuroimage: severe enterovirus encephalitis as a complication of ocrelizumab treatment for multiple sclerosis. Neurology. 2025;104:e213585. PubMed https://doi.org/10.1212/WNL.0000000000213585

8. Banks SA, Poliakov I, Flanagan EP, Toledano M, Nathoo N. Enterovirus encephalitis in people with multiple sclerosis on ocrelizumab: insights from a multicenter case series. Neurol Neuroimmunol Neuroinflamm. 2026;13:e200534. PubMed https://doi.org/10.1212/NXI.0000000000200534

9. Starosta AD, Ehler J, Löffler B, Tannapfel A, Zipprich A, Reuken PA, et al. Echovirus serotype 11 induced sepsis in a young female patient with multiple sclerosis treated with anti-CD20 monoclonal antibody ocrelizumab. Infection. 2025;53:1507–12. PubMed https://doi.org/10.1007/s15010-025-02479-y

10. Stögbauer J, Schegerer V, Berger FK, Schulz-Schaeffer W, Fassbender K, Naumann J, et al. Severe coxsackie virus B5 encephalitis mimics autoimmune limbic encephalitis in a young woman under long-term B-cell depletion with ocrelizumab: a case report. Mult Scler. 2025;31:1258–61. PubMed https://doi.org/10.1177/13524585251322327

11. Mahajan KR, Bittner F, Willis MA, Isada C, Bermel R, Fox RJ, et al. Anti-CD20 associated enterovirus meningoencephalitis: a narrative review with two illustrative cases. Mult Scler J Exp Transl Clin. 2026;12:20552173261471722. PubMed https://doi.org/10.1177/20552173261471722

12. Trentadue T, Martinez Sosa S, Itoh CY, Modi T, Norgan AP, Triplet E, et al. Clinical reasoning: a 30-year-old female patient with multiple sclerosis presenting with rapidly progressive cranial neuropathies, weakness, and ataxia. Neurology. 2025;105:e214429. PubMed https://doi.org/10.1212/WNL.0000000000214429

13. Nel H, Merritt A, Lawson E, Porter M. Disseminated enterovirus infection treated with intravenous immunoglobulin in a patient on maintenance ocrelizumab for multiple sclerosis. BMJ Case Rep. 2025;18:e261915. PubMed https://doi.org/10.1136/bcr-2024-261915

14. Bestwick HMW, Corrigan AE, Baheerathan A, Houlihan CF, Mallon DH, Spillane J, et al. Novel treatment of enterovirus encephalitis associated with B-cell depleting therapy. Clin Infect Practice. 2026;29:100537. https://doi.org/10.1016/j.clinpr.2026.100537

15. Kus S, Creed M, Hernandez-Jones A, Imitola J, Coban H. Enterovirus radiculomyelitis in a relapsing-remitting multiple sclerosis patient (P3–6.002). Neurology. 2024;102:3863. https://doi.org/10.1212/WNL.0000000000205428

16. Kwok S, Jia J, Duggins A, Oon J, Hawke S. Enterovirus infection post Ocrelizumab therapy. In: Abstracts of the 2023 Australian and New Zealand Association of Neurologists (ANZAN) meeting; Hobart, Tasmania, Australia; 2023 May 15–18. Abstract 2676. BMJ Neurology Open. 2024;5. https://doi.org/10.1136/bmjno-2023-ANZAN.85

17. Martin de Frémont G, Chabrolles H, Mirand A, L'Honneur AS, Mélé N, Dunogue B, et al. Severe enterovirus infections in patients with immune-mediated inflammatory diseases receiving anti-CD20 monoclonal antibodies. RMD Open. 2024;10:e004036. PubMed https://doi.org/10.1136/rmdopen-2023-004036

Appendix Table 1. Number of positive specimens tested for enterovirus and parechovirus in Alberta, Canada, 2023–2025*

	No. (%) samples/study year			
Specimen type	2023	2024	2025	Total no. (%)
CSF	15 (35.7)	8 (13.8)	17 (27.4)	40 (24.7)
Blood	0	1 (1.7)	7 (11.3)	8 (4.9)
Heart tissue	0	0	3 (4.8)	3 (1.9)
Mouth swab	14 (33.3)	14 (24.1)	9 (14.5)	37 (22.8)
Nasopharyngeal swab	1 (2.4)	2 (3.4)	2 (3.2)	5 (3.1)
Lip swab	1 (2.4)	0	3 (4.8)	4 (2.5)
Nare swab	1 (2.4)	0	0	1 (0.6)
Skin swab	2 (4.8)	16 (27.6)	18 (29.0)	36 (22.2)
Feces	7 (16.7)	12 (20.7)	1 (1.6)	20 (12.3)
Throat swab	1 (2.4)	4 (6.9)	2 (3.2)	7 (4.3)
Conjunctiva swab	0	1 (1.7)	0	1 (0.6)
Total	42 (25.9)	58 (35.8)	62 (38.3)	162

*CSF, cerebrospinal fluid. Data are for all enterovirus- and parechovirus-positive specimens received during the entire study period.

Appendix Table 2. Annual counts of invasive enterovirus and parechovirus cases according to age group, Alberta, Canada, 2023–2025*

	Age group				
Study year	17–29 y	30–49 y	50–64 y	≥65 y	Total no.
2023, May–Dec	6	8	0	0	14
2024	3	3	1	1	8
2025	7	9	0	1	17
Total	16	20	1	2	39

*Study years 2024 and 2025 were full years (January–December).

Appendix Table 3. Other clinical characteristics of patients with invasive enterovirus and parechovirus infections, Alberta, Canada, 2023–2025*

Characteristic	Not IC, n = 33; 84.6%	IC by ocrelizumab, n = 3; 7.7%	IC for other reasons, n = 3; 7.7%	Total
Lumbar puncture performed	33 (100)	1 (33.3)	3 (100)	37 (94.9)
CSF parameters, median (range) [no. patients]†				
Leukocyte count, $\times 10^6$ cells/L	65 (2–1459) [29]	33	101 (50–880)	NA
Neutrophil count, $\times 10^6$ cells/L	9.4 (0–569.9) [27]	2.0	5.1 (3.5–114.4)	NA
Lymphocyte count, $\times 10^6$ cells/L	24.5 (0.1–1429.8) [27]	29.4	48.5 (45–633.6)	NA
Glucose, mmol/L	3.4 (2.6–4.2) [33]	3.2	3.7 (3.1–3.9)	NA
Protein, g/L	0.45 (0.3–0.86) [33]	0.78	0.69 (0.42–0.88)	N/A
Travel within 6 mo before seeking care	3 (9.1)	0	0	3 (7.7)
Not asked about travel	9 (27.3)	0	0	9 (23.1)
HIV testing done at time of initial care	7 (21.2)	3 (100)	2 (66.7)	12 (30.8)
HIV positive result	0	0	0	0
Head/brain imaging done at time care was sought, CT or MRI scan	25 (75.8)	2 (66.7)	3 (100)	30 (76.9)
Head imaging findings, CT or MRI scan				
No acute intracranial abnormality	25 (100)‡	1 (50)	2 (66.7)	28 (93.3)
Cerebral edema	0	1 (50)	0	1 (3.3)
Mild leptomeningeal enhancement throughout both hemispheres	0	0	1 (33.3)	1 (3.3)

*Values are no. (%) except as indicated. Total no. patients with invasive infection was 39. CSF, cerebrospinal fluid; CT, computed tomography; HIV, human immunodeficiency virus; IC, immunocompromised; MRI, magnetic resonance imaging; NA, not applicable.
†Reference ranges for CSF: leukocyte counts, <5 $\times 10^6$ cells/L; glucose, 2.2–4.4 mmol/L; protein, 0.15–0.45 g/L.
‡One patient initially had a report of a sagging brainstem, which was not observed on follow-up imaging the next day.

Appendix Table 4. Clinical and laboratory characteristics of immunosuppressed patients who had invasive enterovirus infections*

Patient no.†	Immunosuppressive condition‡	Enterovirus positive specimens	Enterovirus syndrome	CSF parameters§	Symptoms	Imaging findings	Cardiac involvement	Enterovirus genotyping outcome
1	Multiple sclerosis (RR) (diagnosed 10 y before), on ocrelizumab for 2 y before first manifestation. Had low IgG levels at initial manifestation (4.92 g/L) and at relapse (6.31 g/L).	CSF: 2 samples collected 293 d apart. Blood: 2 samples collected 294 d apart. NP swab sample was positive for ERV (using RPP) at time of second episode.	Enterovirus encephalitis with relapse 294 d after initial infection diagnosis.	First manifestation: 33 $\times 10^6$ leukocytes/L (89% lymphocytes); protein, 0.78 g/L; glucose, 3.2 mmol/L. Second manifestation: 50 $\times 10^6$ leukocytes/L (90% lymphocytes); protein, 0.42 g/L; glucose, 3.7 mmol/L	Initial episode: brain fog, fatigue, apathy, and depressive catatonia. Relapse: progressive confusion, malaise, and fever. Had contact with a sick household contact before initial manifestation.	MRI of brain: increased FLAIR and T2 signal within the thalami bilaterally on background of demyelinating lesions. No new findings after relapse.	No	Genotype EV-A71 treated with IVIG at the time of both manifestations: 3 doses on first admission (given at 29, 36, and 43 after admission date) and 1 dose on second admission (given 7 d after admission). Patient survived. Previously reported (*9*).
2	Multiple sclerosis (RR) for many years. Started on ocrelizumab 5 y before infection. Had low IgG levels (6.23 g/L) at initial manifestation. No contacts with sick persons before admission.	NP swabs and endotracheal suction were positive for ERV using RPP. Blood and cardiac tissue sample positive for enterovirus.	Gastrointestinal and respiratory illness leading to sepsis and cardiogenic shock, caused by fulminant myocarditis.	ND	Two weeks of ILI and gastritis symptoms that progressed to subsequent respiratory deterioration. No known contact with sick person.	TEE showed biventricular akinesis with an LV ejection fraction of 10%.	Yes, required ECMO	Coxsackie B3 Died as a result of invasive enterovirus infection despite receiving IVIG (1 dose 5 d after admission).
3	Renal transplant 16 y before enterovirus infection. Maintained on prednisone, mycophenolate, and tacrolimus. No contacts with sick persons before disease manifestation.	CSF was positive for enterovirus.	Enterovirus meningitis	Leukocytes, 101 $\times 10^6$ cells/L (48% lymphocytes), protein, 0.69 g/L; glucose, 3.9 mmol/L	Several days of occipital headaches, neck stiffness, fevers, and altered depth perception.	MRI of brain: prominent leptomeningeal enhancement throughout both hemispheres	No	Not typable. Given 1 dose of IVIG 1 d after admission. Patient survived.
4	Crohn's disease, on infliximab for >5 y. Household contact had hand, foot and mouth disease 2 wk before.	CSF was positive for enterovirus.	Enterovirus meningitis	Leukocytes, 880 $\times 10^6$ cells/L (72% lymph); protein, 0.88 g/L; glucose, 3.1 mmol/L	Several days of occipital headache radiating to shoulder, photophobia, nausea with emesis.	MRI of brain: No acute finding.	No	Echovirus-9. No IVIG given. Patient survived.
5	Multiple sclerosis (RR) for 10 y. Began treatment with ocrelizumab ≈5 y before infection. Had low IgG (0.94 g/L) at admission. No contacts with sick persons before admission.	Multiple NP swab samples were positive for ERV. Multiple blood samples taken (viremic for 26 d).	Myocarditis, colitis	ND	Admitted with severe myocarditis. Required ECMO to bridge to cardiac transplant.	TEE showed severe LV dysfunction (10% ejection fraction) and biventricular dysfunction.	Yes	Coxsackie B3. Died as a result of invasive enterovirus infection despite receiving 2 doses of IVIG (given at 43 and 71 d after admission date).

Patient no.†	Immunosuppressive condition‡	Enterovirus positive specimens	Enterovirus syndrome	CSF parameters§	Symptoms	Imaging findings	Cardiac involvement	Enterovirus genotyping outcome
		Cardiac tissue was positive for enterovirus.						
6	B cell lymphoma treated with chemotherapy and had 6 doses of rituximab in previous 3 y. No contact with sick persons before admission.	CSF was positive for enterovirus.	Enterovirus meningitis	Leukocytes, 114 × 10^6 cells/L (95% lymph); protein, 0.83 g/L, glucose, 2.1 mmol/L	Had 2-wk history of headache, neck and shoulder pain, gait unsteadiness, and light headedness.	Neither CT scan of head or MRI of brain showed acute radiographic abnormalities.	No	Not typable due to low viral load in sample. No IVIG administered. Patient survived.

*CSF, cerebrospinal fluid; CT, computed tomography; ECMO, extra corporeal membrane oxygenation; ERV, enterovirus and rhinovirus infection; EV-A71, enterovirus A71; FLAI, fluid-attenuated inversion recovery; ILI, influenza-like illness; IVIG, intravenous immunoglobulin; LV, left ventricle; MRI, magnetic resonance imaging; ND, not done; NP, nasopharyngeal; RPP, respiratory pathogen panel (virus panel that has a targets for ERV; but cannot distinguish between the 2 viruses); RR, relapsing/remitting; TEE, transesophageal echocardiogram.

†Four (66.7%) of 6 patients were female. Median age for all 6 patients was 35.5 y (mean 39.2; range 29–67). Age recorded is at time of invasive enterovirus diagnosis.

‡One fatal ocrelizumab-treated patient was found to be heterozygous for a *TNFRSF13B* gene variant causing the C104R mutation (patient no. 5) and had no circulating B cells at the time of disease manifestation. The surviving ocrelizumab-treated patient (patient no. 1) tested negative for this variant. The remaining patients in this table were not tested for this genetic variant by their clinicians upon chart review.

§Reference ranges for leukocytes, glucose, and protein in CSF are in Appendix Table 3.

Appendix Table 5. Virus genotyping results for invasive enterovirus isolates collected each study year, Alberta, Canada*

Virus	2023	2024	2025
Coxsackievirus B3	NA	NA	2
Coxsackievirus B4	3	NA	NA
Coxsackievirus B5	1	NA	NA
Echovirus 6	NA	NA	1
Echovirus 9	NA	1	2
Echovirus 18	NA	1	1
Echovirus 30	NA	NA	5
Enterovirus A71	NA	1	NA

*NA, not applicable. Eighteen isolates (4 of 14 in 2023; 3 of 8 in 2024; 11 of 17 in 2025) could be amplified for genotyping from a total of 39 unique patients.

Appendix Table 6. GenBank accession numbers of submitted sequences*

Study number†	Sample type	GenBank accession no.
2023_D001	CSF	PZ914344
2023_D107	CSF	PZ914345
2023_D111	CSF	PZ914348
2023_D114	CSF	PZ914346
2024_D124	CSF	PZ914355
2024_D209	CSF	PZ914357
2024_D265	CSF	PZ914347
2025_D001	CSF	PZ914351
2025_D012	Blood	PZ914341
2025_D017	CSF	PZ914356
2025_D102	CSF	PZ914354
2025_D133	CSF	PZ914352
2025_D163	Blood	PZ914343
2025_D176	CSF	PZ914342
2025_D180	CSF	PZ914358
2025_D204	CSF	PZ914349
2025_D206	CSF	PZ914350
2025_D271	CSF	PZ914353

*CSF, cerebrospinal fluid.
†Study numbers include the year the isolate was collected.

Appendix Table 7. Reported cases of invasive enterovirus infection in patients receiving ocrelizumab*

Citation	Clinical information	Virus	Treatment and outcome
(*6*)	44-y-old female with RR-MS, on ocrelizumab for 4.5 y with fulminant hepatitis and peak ALT at 6241 U/L. Plasma and liver biopsy samples were positive for enterovirus RNA.†	Echovirus 25	Required liver transplant and withdrawal of ocrelizumab. Patient survived.
(*7*)‡	34-y-old male with RR-MS, on ocrelizumab for 32 mo. Had encephalitis/meningoencephalitis along with a chronic decline over several weeks. Enterovirus RNA detected in blood and CSF.	EV-A71	Ocrelizumab was discontinued. Received IVIG × 3 wk and had clinical improvement. Patient survived.
(*8*)	Case series (n = 5) of persons with MS (median age, 34 y) receiving ocrelizumab (median, 3 y) who had encephalitis. One patient had concurrent myocarditis.	EV-A71; Coxsackievirus B3	IVIG was given to 3 case-patients. One case-patient fully recovered and 4 had residual cognitive/gait deficits. Ocrelizumab was stopped. All survived.
(*9*)	20-y-old female with MS who had sepsis, hepatitis, enterocolitis, pancreatitis, pneumonia, and hearing loss. Detectable enterovirus RNA was found in multiple specimens, including blood and pleural fluid.	Echovirus 11	Ocrelizumab was stopped and IVIG was administered × 3 wk + fluoxetine. Patient survived.
(*10*)	33-y-old female with RR-MS, on ocrelizumab for 4 y who had severe limbic syndrome. Treated for limbic encephalitis. Broad range testing found enterovirus RNA in CSF.	Coxsackievirus B5	Treated with ribavirin, favipravir, IVIG × 4 wk. Patient survived but had severe residual cognitive deficits.
(*11*)	33-y-old male with MS, on ocrelizumab for 4 y who had symptoms of aseptic meningitis complicated by nystagmus and diplopia. CSF was positive for enterovirus RNA. Disease progressed to encephalopathy with expressive aphasia along with myoclonus and dysautonomia.	Enterovirus, not typed	IVIG and methylprednisolone × 5 d followed by pocapavir × 14 d. Ocrelizumab was discontinued. Patient survived.
(*12*)	30-y-old female, 4 months postpartum, who had diplopia, dysarthria, upper extremity diplegia, and nausea. CSF eventually found to contain enterovirus RNA after shotgun sequencing.	Enterovirus	Daily IVIG × 5 d. Three wk later, diplopia resolved but with residual findings of ocular flutter, ataxic dysarthria, and upper limb dysmetria. Patient chose to restart ocrelizumab. Patient survived.
(*13*)	Female in her 4th decade of life, on ocrelizumab for 6 y for RR-MS, who initially had respiratory infection caused by enterovirus and rhinovirus. PUO work up post–respiratory tract infection found persistent enterovirus viremia and enterovirus in feces.	Enterovirus, could not be amplified for sequencing.	Treated with IVIG (2 doses 2 wk apart) with resolution of symptoms. Patient survived.
(*14*)	41-y-old female with MS on ocrelizumab had vertigo, oscillopsia, dysarthria, dysphagia, and meningoencephalitis and was found to have rhombencephalitis. CSF testing was unremarkable. Enterovirus RNA was detected in blood and feces.	Enterovirus, description of further testing not provided.	Treated with IVIG, steroids, and antivirus drugs. Two doses of IVIG was given 2 wk apart. At 3 wk, a combination of remdesivir, fluoxetine, and ribavirin was given. Patient showed slow improvement and good outcome. Patient survived.
(*15*)§	31-y-old male with MS on ocrelizumab had fever, headache, urinary retention, and bilateral lower limb weakness. NP swab and CSF samples had enterovirus RNA.	Enterovirus, no further details given.	5-d course of IVIG, fluoxetine, and high dose steroids. Patient survived.
(*16*)§	25-y-old female with MS had meningoencephalitis and had experienced hand, foot and mouth disease 2 wk before encephalitis onset. Enterovirus RNA was detected in CSF.	Enterovirus, no further details given.	Supportive therapy provided without steroids, IVIG, or antivirus drugs. Patient survived.
(*17*)	Review of invasive enterovirus infections in National Reference Center in 2 cities in France. Nine patients were identified, of which 1 was using ocrelizumab.	Invasive enterovirus, no typing data available specifically for that patient.	Not reported.

*ALT, alanine aminotransferase; CSF, cerebrospinal fluid; EV-A71, enterovirus A71; IVIG, intravenous immunoglobulin; MS, multiple sclerosis; NP, nasopharyngeal; PUO, pyrexia of unknown origin; RR-MS, relapsing/remitting multiple sclerosis.
†Enterovirus RNA was detected by using PCR.
‡This imaging case report describes 1 of the patients included in our study from 2024.
§Abstract from published meeting proceedings.

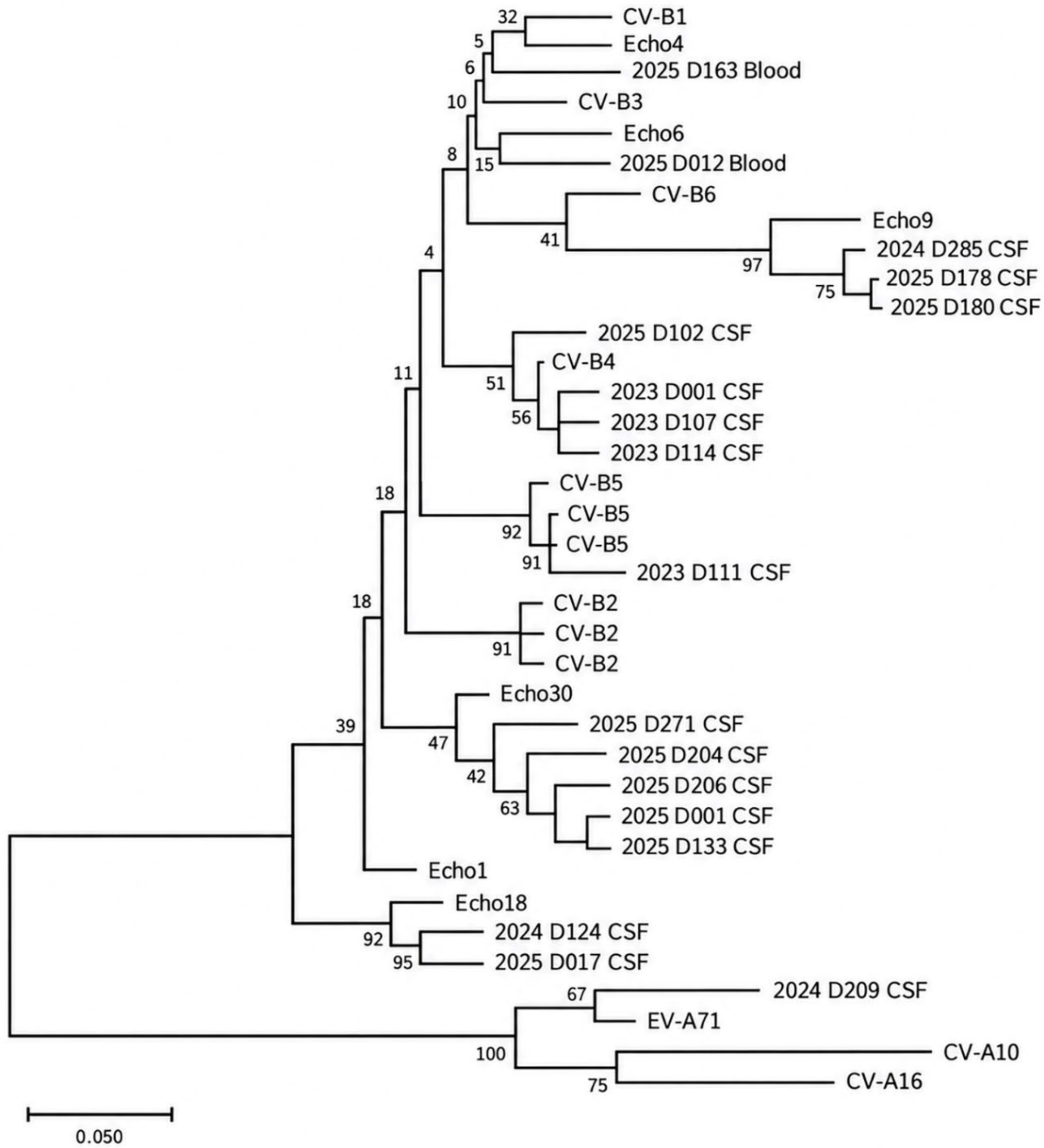

Appendix Figure. Phylogenetic analysis of the virus protein 2 gene region of enterovirus in clinical isolates. Taxa in bold font represent isolates from adult patients typed in this study, labeled as year, D no. (study no.) and specimen type. Main specimen types were blood and CSF. Study numbers were assigned for the purposes of this study. Reference strains are labeled according to virus type. CSF, cerebrospinal fluid; CV, coxsackievirus; Echo, echovirus; EV, enterovirus. Scale bar indicates nucleotide substitutions per site.
